# Association between Serum IL-37 and Spinal Cord Injury: A Prospective Observational Study

**DOI:** 10.1155/2020/6664313

**Published:** 2020-12-10

**Authors:** Yuanzhen Chen, Dandan Wang, Shengnan Cao, Guangjian Hou, Hong Ma, Bin Shi

**Affiliations:** ^1^Neck-Shoulder and Lumbocrural Pain Hospital, Shandong First Medical University & Shandong Academy of Medical Sciences, Jinan, 250014 Shandong Province, China; ^2^Shandong First Medical University, Jinan, 250014 Shandong Province, China; ^3^School of Acupuncture-Tuina, Shandong University of Traditional Chinese Medicine, Jinan, 250355 Shandong Province, China; ^4^Foshan Traditional Chinese Medicine Hospital, Foshan, 528000 Guangdong Province, China

## Abstract

**Objective:**

Interleukin-37 (IL-37) is a new cytokine that naturally inhibits inflammation. Inflammation plays an important role in acute spinal cord injury (SCI). The purpose of this study is to check whether serum IL-37 can be used as a clinical predictor of SCI.

**Methods:**

All subjects underwent venipuncture within 24 hours of enrollment to obtain peripheral blood and then centrifuged to obtain serum. The concentration of serum IL-37 was determined by enzyme-linked immunosorbent assay (ELISA). One month after the injury, the American Spinal Cord Injury Association (ASIA) impairment scale was used for neurological examination.

**Results:**

A total of 148 people were included in the study, including 52 normal controls (NC) and 96 patients with acute SCI within 24 hours of onset. The comparison of clinical baseline data (age, gender, BMI: body mass index, smoking, alcohol drinking, CHD: coronary heart disease, HBP: high blood pressure, and DM: diabetes mellitus) between the two groups was not statistically significant (*p* > 0.05). However, the serum IL-37 concentration of SCI patients was significantly higher than that of the NC group, and the difference was statistically significant (*p* < 0.001). And with the aggravation of SCI grade, the level of IL-37 increased significantly (*p* < 0.05). Pearson correlation analysis further showed that serum IL-37 concentration is negatively correlated with AISA motor score (*r* = −0.327, *p* < 0.05).

**Conclusion:**

The serum IL-37 concentration of SCI patients is significantly increased, and it is closely related to the recovery of motor function. We proved for the first time that serum IL-37 has prognostic value in patients with SCI. In addition, serum IL-37 may be used as a prognostic biomarker for SCI.

## 1. Introduction

Spinal cord injury (SCI) refers to spinal cord injury that temporarily or permanently changes the function of the spinal cord [1]. Every year, about 200,000 people in the world have to face the consequences of SCI [2]. In particular, the impairment of motor function prevents the patients from being completely independent of function, which brings a heavy burden to the family and society [3]. In the United States, there are approximately 17,000 new cases of SCI recorded each year [4]. Therefore, it is necessary to find biomarkers of SCI in order to better understand the potential molecular mechanisms of neurological damage after SCI and develop new treatment strategies.

Kumar et al. first discovered and identified the existence of interleukin-37 (IL-37) through computational sequence analysis in 2000 [5]. It was originally named IL-1H4 or IL-1F7. Later, Nold et al. found that it has the effect of suppressing the innate immune response and officially renamed it IL-37 [6]. Recently, IL-37 is considered to play an important regulatory role in the occurrence and development of various inflammatory diseases, autoimmune diseases, and vascular diseases [7–9]. However, the pathophysiological mechanism of IL-37 in the body has not yet been fully elucidated.

Animal experiments have suggested that IL-37 may be involved in the pathogenesis of SCI [10]. The purpose of our study is to check whether serum IL-37 can be used as a clinical predictor of SCI. If the relationship between IL-37 and SCI is confirmed, it will provide new possibilities for the prevention and treatment of SCI.

## 2. Methods

### 2.1. Study Population

Patients with acute SCI who attended Neck-Shoulder and Lumbocrural Pain Hospital, Shandong First Medical University & Shandong Academy of Medical Sciences from July 2017 to June 2020 were included in the study. The diagnosis of SCI is based on clinical manifestations and impact studies and is made by experienced clinicians [11]. The entry criteria of the SCI group: meet the diagnostic criteria of SCI; the onset is within 24 hours; the vital signs are stable. Exclusion criteria for the SCI group: accompanied by traumatic brain injury; severe infectious diseases; tumors; autoimmune diseases; take recent antibiotics or corticosteroids; multiple system dysfunction; failure to cooperate with examinations. In addition, volunteers matched with age and sex were selected as the normal control group (NC). A total of 148 subjects were included in this study, including 52 in the NC group and 96 in the SCI group. All subjects or guardians were informed of this study and signed a consent form. This study complied with the Declaration of Helsinki and was approved by the ethics committee of Neck-Shoulder and Lumbocrural Pain Hospital, Shandong First Medical University & Shandong Academy of Medical Sciences.

### 2.2. Clinical Baseline Data

The clinical baseline data were collected when the subjects were enrolled. The clinical baseline data is recorded by specialized clinicians. The recorded clinical baseline data include demographic data, living habits, and past medical history. Demographic data includes age, gender, and body mass index (BMI). Living habits include smoking and alcohol drinking. Past medical history includes coronary heart disease (CHD), hypertension (HBP), and diabetes (DM).

### 2.3. Determination of Serum IL-37 Concentration

Within 24 hours of admission, 5 ml of fasting blood from all subjects was collected into a test tube with a lid. After collecting the whole blood, the peripheral blood is allowed to coagulate at room temperature for 20 minutes. The peripheral blood was then centrifuged at 2,000 × g for 15 minutes in a refrigerated centrifuge. After centrifugation, immediately transfer the upper serum to a clean polypropylene tube. If the serum is not analyzed immediately, the serum should be divided into 0.5 ml aliquots and stored at -80°C. The serum IL-37 concentration was determined using a sandwich enzyme-linked immunosorbent assay (ELISA). The ELISA experiment uses the commercialized Human IL-37 ELISA Kit (Abcam, Cambridge, MA, USA). The experimental procedures of ELISA are based on the reagent instructions and previous reports [12]. The experimental method is as follows: add 100 *μ*L standard or sample to appropriate wells incubated at 37°C for 90 minutes; discard plate content and then add 100 *μ*L biotinylated antibody into all wells incubated at 37°C for 60 minutes. Wash each well three times with 300 *μ*L 0.01 M PBS; add 100 *μ*L ABC working solution incubated at 37°C for 30 minutes; wash each well five times with 300 *μ*L 0.01 M PBS and add 90 *μ*L of prepared TMB incubated at 37°C in dark for 30 minutes; add 100 *μ*L TMB Stop Solution and read OD at 450 nm within 30 minutes. Finally, the concentration of serum IL-37 was calculated by the standard curve.

### 2.4. Cord Injury Association Impairment Scale Evaluation

Cord Injury Association (ASIA) impairment scale evaluation is considered the international standard for neurological classification of spinal cord injury [13]. The ASIA impairment scale is used by ASIA as a general classification tool for spinal cord injuries, used to standardize sensory and motor functions, and is widely used in many countries around the world. According to the ASIA Impairment Scale, SCI can be divided into five levels: Grade A, complete injury. No motor or sensory function is preserved in the sacral segments S4 or S5; Grade B, sensory incomplete. Sensory but not motor function is preserved below the level of injury, including the sacral segments; Grade C, motor incomplete. Motor function is preserved below the level of injury, and more than half of muscles tested below the level of injury have a muscle grade less than 3; Grade D, motor incomplete. Motor function is preserved below the level of injury and at least half of the key muscles below the neurological level have a muscle grade of 3 or more; Grade E, normal. No motor or sensory deficits but deficits existed in the past. The total scores of motor function and sensory function evaluation were 100 and 224 points, respectively [14–16]. ASIA impairment scale evaluation is done by professionally trained physicians, and the physicians do not know the grouping and clinical baseline data of the subjects.

### 2.5. Statistical Analysis

All statistical calculations are performed using SPSS 20.0 (SPSS Inc., Chicago, IL, USA). Continuous variables are described by mean ± standard deviation (SD), and categorical variables are described by numbers. Differences between groups were analyzed by one-way analysis of variance (ANOVA), and post hoc Newman-Keuls test was used for multiple groups. The correlation between variables is evaluated using the Pearson correlation coefficient. The statistical significance is set to 0.05.

## 3. Results

### 3.1. Clinical Baseline Data

The results of the clinical baseline data are shown in Table 1. The clinical baseline data of this study include age, gender, BMI, smoking, alcohol drinking, CHD, HBP, and DM. The results showed that there was no statistical difference between the clinical baseline data of the NC group and the SCI group (*p* > 0.05).

### 3.2. Serum Concentration of IL-37

**Figure 1:**
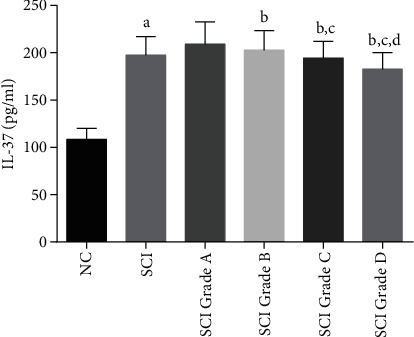
Serum concentration of IL-37. NC: normal controls; SCI: spinal cord injury. Compared with NC, ^a^*p* < 0.001; compared with SCI Grade A, ^b^*p* < 0.05; compared with SCI Grade B, ^c^*p* < 0.05; compared with SCI Grade C, ^d^*p* < 0.05.

The concentration of serum IL-37 was determined by ELISA. The results of the serum concentration of IL-37 are shown in Figure 1 and Table 2. The results showed that the serum IL-37 concentrations of NC group and SCI group were (108.46 ± 11.74) pg/ml and (197.31 ± 19.68) pg/ml, respectively, and there was a statistical difference between the two (*p* < 0.05). SCI patients are divided into four grades according to the AISA Impairment Scale, namely, Grade A, Grade B, Grade C, and Grade D. The serum IL-37 concentrations of patients with SCI Grade A, Grade B, Grade C, and Grade D were (209.16 ± 23.3) pg/ml, (202.75 ± 20.74) pg/ml, (194.52 ± 17.41) pg/ml, and (182.81 ± 17.22) pg/ml, and there are statistical differences among the four groups (*p* < 0.05).

### 3.3. Pearson Correlation Analysis

**Figure 2:**
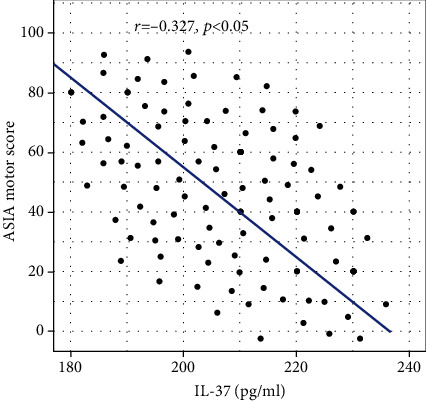
Pearson correlation analysis between IL-37 and ASIA motor score in SCI. ASIA: American Spinal Cord Injury Association; SCI: spinal cord injury.

The results of Pearson correlation analysis between IL-37 and ASIA motor score are shown in Figure 2. The results showed that in SCI patients, serum IL-37 concentration and ASIA motor score were negatively correlated (*r* = −0.327, *p* < 0.05).

## 4. Discussion

The main finding of this study is that the serum IL-37 concentration is elevated in SCI patients, and it is negatively correlated with ASIA motor scores. Further research found that serum IL-37 can be used as a potential biomarker for the diagnosis of SCI. For the first time, we have confirmed the correlation between IL-37 and the prognosis of motor function in SCI patients.

The mechanism of SCI is complicated. In the early stages of SCI, mechanical damage causes local neurons and glial cells to die within minutes to hours. The subsequent delayed injury mainly manifested as neuronal and glial cell apoptosis and increased permeability of the blood-spinal cord barrier and neuroinflammatory response [17–19]. Neuroinflammation is an important mechanism of SCI, which can worsen the prognosis, so it is necessary to clarify the inflammatory mechanism of SCI.

IL-37 is a unique member of the IL-1 cytokine family, and its function is a natural inhibitor of inflammation and immune response [20]. Immune cells and nonimmune cells produce IL-37 precursors after proinflammatory stimulation, which is then activated and lysed by caspase-1 to transform into mature IL-37 and transfer to the nucleus, thereby inhibiting the transcription of proinflammatory genes [21, 22]. IL-37 is involved in the inflammatory process of a series of diseases, such as atherosclerosis, asthma, inflammatory bowel disease, psoriasis, rheumatoid arthritis, systemic lupus erythematosus, and ocular inflammatory diseases [7]. Interestingly, studies have pointed out that IL-37 is involved in the pathogenesis of multiple sclerosis, a neurological disease [23]. Iranian scholars found that the serum IL-37 concentration in patients with multiple sclerosis and optic neuromyelitis increased, suggesting that IL-37 is involved in its pathophysiological mechanism [24]. There are also animal experiments that show that the secretions of human periodontal ligament cells pretreated with hypoxia can have anti-inflammatory effects through IL-37 in multiple sclerosis experimental models [25]. The role of IL-37 in nerve injury-related diseases is gradually receiving attention from basic to clinical.

IL-37 may be one of the potential mechanisms involved in the pathogenesis of SCI. Marina Coll-Miró and his colleagues found that the SCI model made using IL-37 transgenic mice showed lower levels of proinflammatory factor secretion and less neurological damage. At the same time, exogenous administration of recombinant IL-37 can reduce the symptoms of SCI and further enhance IL-37's neuroprotective effect on SCI injury [10]. In order to further explore the neuroprotective mechanism of IL-37 on SCI, researchers from many countries have conducted a series of studies in a variety of genetically modified animals. Studies have proved that the translocation of IL-37 to the nucleus is not necessary for the neuroprotection of SCI, but the signal transduction of IL-37 outside the cell plays a decisive role [19]. However, we first confirmed the correlation between IL-37 and SCI in a clinical study.

Our research has some limitations. First, our sample size is small. Second, we are a single-center study, and its results may not be applicable to people from other regions or races. Third, we did not conduct dynamic monitoring of serum IL-37 nor did we conduct long-term follow-up of patients with SCI. Nevertheless, as far as we know, our study reported for the first time that IL-37 is involved in the pathogenesis of clinical SCI patients, which has important inspiration for the prevention and treatment of SCI.

## 5. Conclusions

Our research shows that the serum IL-37 of patients with SCI is significantly increased, and it is obviously related to the grades of SCI. That is, the more severe the SCI condition, the higher the serum SCI level. At the same time, serum IL-37 was significantly negatively correlated with the ASIA motor score of SCI, suggesting that IL-37 in the acute phase can predict the prognosis of SCI. Finally, serum IL-37 may be a potential biomarker for predicting SCI.

## Figures and Tables

**Table 1:** Clinical baseline data.

	NC (*n* = 52)	SCI (*n* = 96)	*p*
Age (years)	49.38 ± 5.21	50.17 ± 5.09	0.373
Gender (male/female)	31/21	60/36	0.731
BMI (kg/m^2^)	24.75 ± 1.43	24.81 ± 1.50	0.814
Smoking (*n*)	31	54	0.693
Alcohol drinking (*n*)	36	61	0.487
CHD (*n*)	4	7	0.929
HBP (*n*)	8	17	0.719
DM (*n*)	7	11	0.722

Abbreviation: NC: normal controls; SCI: spinal cord injury; BMI: body mass index; CHD: coronary heart disease; HBP: high blood pressure; DM: diabetes mellitus.

**Table 2:** Serum concentration of IL-37.

	NC	SCI				
		Total	Grade A	Grade B	Grade C	Grade D
IL-37 (pg/ml)	108.46 ± 11.74	197.31 ± 19.68^a^	209.16 ± 23.35	202.75 ± 20.74^b^	194.52 ± 17.41^b,c^	182.81 ± 17.22^b,c,d^

Abbreviation: NC: normal controls; SCI: spinal cord injury; IL-37: interleukin 37; ASIA: American Spinal Cord Injury Association; SCI: spinal cord injury; IL-37: interleukin 37. Compared with NC, ^a^*p* < 0.001; compared with SCI Grade A, ^b^*p* < 0.05; compared with SCI Grade B, ^c^*p* < 0.05; compared with SCI Grade C, ^d^*p* < 0.05.

## Data Availability

The data used to support the findings of this study are available from the corresponding author upon request.
